# Enhancement of cardiac lymphangiogenesis by transplantation of CD34^+^VEGFR-3^+^ endothelial progenitor cells and sustained release of VEGF-C

**DOI:** 10.1007/s00395-019-0752-z

**Published:** 2019-10-06

**Authors:** Hai-feng Zhang, Yong-li Wang, Yu-zhen Tan, Hai-jie Wang, Ping Tao, Pei Zhou

**Affiliations:** Department of Anatomy, Histology and Embryology, Shanghai Medical School of Fudan University, 138 Yixueyuan Road, Shanghai, 200032 People’s Republic of China

**Keywords:** Lymphangiogenesis, Lymphatic endothelial progenitor cells, VEGF-C, Self-assembling peptide, Myocardial infarction

## Abstract

Impairment of cardiac lymphatic vessels leads to cardiac lymphedema. Recent studies have suggested that stimulation of lymphangiogenesis may reduce cardiac lymphedema. However, effects of lymphatic endothelial progenitor cells (LEPCs) on cardiac lymphangiogenesis are poorly understood. Therefore, this study investigated effectiveness of LEPC transplantation and VEGF-C release with self-assembling peptide (SAP) on cardiac lymphangiogenesis after myocardial infarction (MI). CD34^+^VEGFR-3^+^ EPCs isolated from rat bone marrow differentiated into lymphatic endothelial cells after VEGF-C induction. VEGF-C also stimulated the cells to incorporate into the lymphatic capillary-like structures. The functionalized SAP could adhere with the cells and released VEGF-C sustainedly. In the condition of hypoxia and serum deprivation or abdominal pouch assay, the SAP hydrogel protected the cells from apoptosis and necrosis. At 4 weeks after intramyocardial transplantation of the cells and VEGF-C loaded with SAP hydrogel in rat MI models, cardiac lymphangiogenesis was increased, cardiac edema and reverse remodeling were reduced, and cardiac function was improved significantly. Delivery with SAP hydrogel favored survival of the engrafted cells. VEGF-C released from the hydrogel promoted differentiation and incorporation of the cells as well as growth of pre-existed lymphatic vessels. Cardiac lymphangiogenesis was beneficial for elimination of the inflammatory cells in the infarcted myocardium. Moreover, angiogenesis and myocardial regeneration were enhanced after reduction of lymphedema. These results demonstrate that the combined delivery of LEPCs and VEGF-C with the functionalized SAP promotes cardiac lymphangiogenesis and repair of the infarcted myocardium effectively. This study represents a novel therapy for relieving myocardial edema in cardiovascular diseases.

**Electronic supplementary material:**

The online version of this article (10.1007/s00395-019-0752-z) contains supplementary material, which is available to authorized users.

## Introduction

Myocardial infarction (MI) is a common cardiac emergency with the potential for substantial morbidity and mortality [2]. After occlusion of a coronary artery or its branch, necrosis of myocardium triggers local inflammation, scar formation and remodeling of the ventricular wall. The patients die of heart failure or arrhythmia finally. Regeneration of the post-infarct myocardium and angiogenesis are vital processes in repair of the infarcted myocardium. However, physiopathologic impacts of cardiac lymphangiogenesis on prognosis of patients with MI have been ignored. There are the subendocardial, myocardial and subepicardial networks of the lymphatic capillaries. Lymph flow in cardiac chambers is from the endocardium to the epicardium. Ultimately, the subepicardial lymphatics join to form the left and right collecting trunks which pass along the left and right coronary vessels to drain to the regional mediastinal lymph nodes [25, 29]. The cardiac lymphatic vessels drain excessive protein and fluid from the extracellular spaces, and transport leukocytes and antigen-presenting cells from the inflammatory tissue. Impairment of lymphatic drainage results in cardiac edema, inflammation and fibrosis post-MI [14]. In addition, lymphostasis causes injury of the conducting tissue, triggering arrhythmia possibly [27]. Noteworthily, blood microvascular hyperpermeability induced by ischemia and inflammation is also involved in cardiac edema. Lymph flow rate is almost doubled in canine model of acute myocardial ischemia [9]. Although increase in the number of lymphatic vessels in the peri-infarcted region is a positive response post-MI [19, 20], remodeling of epicardial precollector and collector lymphatics occurred, leading to reduction of cardiac lymphatic transport capacity [12]. Thus, mild lymphangiogenesis at the peri-infarct region is insufficient for alleviating severe cardiac edema effectively. Moreover, lymphangiogenesis lags behind angiogenesis during myocardial remodeling [15]. Therefore, promoting lymphangiogenesis is considered as a potential strategy for resolution of insufficient cardiac lymphatic drainage.

In recent years, more and more attention has been focused on stimulating lymphangiogenesis to improve lymphatic drainage of the infarcted myocardium [4]. Transplantation of mesenchymal stem cell-loaded cardiac patch on the epicardium with MI enhanced lymphangiogenesis at the infarct and peri-infarct regions [46]. Epicardium-derived adrenomedullin contributed to lymphangiogenesis post-MI. Overexpression of adrenomedullin led to increased lymphangiogenesis, decreased myocardial edema and improvement in cardiac function [42]. With intraperitoneal injection of vascular endothelial growth factor C (VEGF-C), lymphangiogenic response surrounding the injured myocardium was augmented, and cardiac function was improved significantly [20]. In the models of MI or myocardial ischemia and reperfusion, lymphangiogenesis augmented with VEGF-C delivered by gelatin hydrogel placed on the epicardium promoted reduction of myocardial edema, fibrosis and cardiac dysfunction [37]. Intramyocardial delivery of VEGF-C using albumin–alginate microparticles accelerated lymphangiogenesis and limited remodeling of lymphatic vessels post-MI. Myocardial fluid balance was improved, and cardiac inflammation, fibrosis and dysfunction were attenuated [12]. Stimulation of cardiac lymphangiogenesis with intraperitoneal injection of VEGF-C improved clearance of the acute inflammatory response post-MI by trafficking immune cells to the mediastinal lymph nodes [44]. Nevertheless, strategies of targeting lymphangiogenesis such as VEGF-C administration need to be optimized for repairing the infarcted myocardium effectively.

It is now well established that lymphangiogenesis occurs at the pre-existed lymphatic vessels through proliferation, migration and tube formation of the endothelial cells. However, lymphatic endothelial progenitor cells (LEPCs) have not been recognized as contributors to developmental and postnatal lymphangiogenesis until recent years [17, 32]. Endothelial progenitor cells were detected at the growing lymphatic vessels in the cornea of mouse treated with irradiation [34] and the transplanted human kidney [18]. Bone marrow-derived podoplanin^+^ cells could participate in lymphatic neovascularization under inflammatory and tumorigenic conditions [21]. Moreover, podoplanin^+^ cells were involved in growth of lymphatic vessels post-MI [6]. After stimulation with growth factors, some CD34^+^ EPC-derived cells became positive for lymphatic vessel endothelial hyaluronan receptor 1 (LYVE-1) [30]. CD34^+^CD133^+^VEGFR-3^+^ (vascular endothelial growth factor receptor 3) cells from fetal liver could differentiate towards lymphatic and vascular endothelial cells under incubation with endothelial cell medium [35]. CD34^+^VEGFR-3^+^ EPCs isolated from human umbilical cord blood expressed CD133 and differentiated into lymphatic endothelial cells in condition of VEGF-C induction. The differentiated cells could organize into lymphatic-like capillaries in three-dimensional collagen gel and Matrigel [40]. Because the number and function of EPCs in some patients with cardiovascular diseases are reduced as a consequence of exposure to risk factors, it is necessary to transplant autologous or allogenic cells to promote neovascularization for cardiac repair [36, 49]. Nevertheless, effectiveness of LEPC transplantation on promoting lymphangiogenesis of the infarcted myocardium is unknown.

This investigation was designed to examine participation of the implanted LEPCs in cardiac lymphangiogenesis and lymphangiogenic effects of VEGF-C released from self-assembly peptide (SAP). Here, we demonstrate that CD34^+^VEGFR-3^+^ EPCs isolated from bone marrow can differentiate towards lymphatic endothelial cells and incorporate into the lymphatic vessels. SAP-released VEGF-C accelerates differentiation of the cells as well as stimulating growth of the pre-existed lymphatic vessels effectively. Our work is the first evidence that cardiac edema and fibrosis are attenuated significantly after delivery of LEPCs and VEGF-C with the functionalized SAP.

## Methods

### Isolation and identification of CD34^+^VEGFR-3^+^ endothelial progenitor cells

Male Sprague–Dawley (SD) rats (2‒3 weeks, 30‒50 g) were euthanized with ketamine (80 mg/kg). The protocol was permitted by the law of the People’s Republic of China on the Protection of Wildlife and approved by the Institutional Animal Care Committee of Fudan University. Bone marrow in femurs and tibias was harvested by flushing with 0.01 M phosphate-buffered saline (PBS) supplemented with 5 mM ethylene diamine tetraacetic acid (EDTA). The mononuclear cells in bone marrow were obtained using gradient centrifugation with Percoll solution (GE Healthcare, Uppsala, Sweden). Then, CD34^+^VEGFR-3^+^ EPCs were sorted from the mononuclear cells using a fluorescence-activated cell sorter (FACS; Beckman Coulter, Fullerton, CA, USA) according to the proceedings previously described [45]. Expression of CD45, CD11b and CD68 in the sorted cells were analyzed with flow cytometry (FACSCalibur, Becton–Dickinson, San Jose, CA, USA). Prox1 expression in the sorted cells was examined with immunostaining. The sorted cells were seeded in gelatin-coated dishes at density of 3 × 10^5^/mL and incubated in Dulbecco’s modified Eagle’s medium (DMEM; Thermo Fisher Scientific, Waltham, MA, USA) supplemented with 15% fetal bovine serum (FBS), 100 U/mL penicillin, 100 μg/mL streptomycin (Thermo Fisher Scientific) and 10 ng/mL recombinant human VEGF-C (100-20C, PeproTech, Rocky Hill, NJ, USA) at 37 °C in a humidity atmosphere of 5% CO_2_. At 2 weeks after inducing with VEGF-C, the endothelial feature of the monolayer of the differentiated cells was examined with silver nitrate staining [39]. Differentiation of CD34^+^VEGFR-3^+^ EPCs into lymphatic endothelial cells was identified by acquisition of 5′-nucleotidase (5′-Nase), VEGFR-3 and LYVE-1 expression.

### Tube formation assay

Property of CD34^+^VEGFR-3^+^ EPCs (hereafter termed “LEPCs”) in participating in lymphangiogenesis was assessed with tube formation in Matrigel. Lymphatic endothelial cells (LECs) were isolated from the thoracic ducts of SD rats. After the surrounding connective tissue was removed, the thoracic duct was cut into 1 mm^2^ pieces. Then, the tissue pieces were implanted into gelatin-coated culture dishes and incubated with DMEM containing 10% FBS and 10 ng/mL VEGF-C. The endothelial side of the pieces was down. When LECs migrated from the pieces and grew to colonies, the tissue pieces were removed out. LECs were passaged at a ratio of 1:2 till the cells grew to monolayer. The cells of the third to fifth passages were used. The isolated LECs were identified with LYVE-1 immunostaining and silver nitrate staining. In tube formation experiment, mixture of Matrigel (Corning, Bedford, MA, USA) and DMEM (3:2) was dropped into 24-well plate and allowed to gelate at 37 °C for 1 h. Then, 5 × 10^4^ LECs were seeded on the gel and incubated in the medium supplemented with 1% FBS for 2 h. After capillary-like structures were formed, 1 × 10^4^ LEPCs labeled with Dil (Beyotime Biotech, Haimen, Jiangsu, China) were added and incubated continually for 2 h. Incorporation of LEPCs into the capillary-like structures was examined with a fluorescent microscope. In VEGF-C group, the cells were treated with 10 ng VEGF-C.

### Atomic force and scanning electron microscopies

SAP (AcN-RADARADARADARADARGDS-CONH_2_) was custom-synthesized by Top-peptide Biotechnology (Shanghai, China). RGDS was designed as cell adhesion motif. Powder of the fabricated SAP was dissolved in sterile distilled water at concentration of 1% (10 mg/mL) before using. For assessing self-assembling of the peptide, SAP solution was diluted to concentration of 0.01%. 5 μL diluted samples were dropped onto a freshly cleaved mica surface. Then, the surface was gently rinsed twice with distilled water and air-dried for 30 min at room temperature. The nanofibers assembled by SAP were examined with an atomic force microscope (Dimension Icon, Bruker, USA). In scanning electron microscopy, the SAP solution was mixed with 0.01 M PBS (1:1) and then sonicated for 30 min. The mixture was dropped onto a cover slip and allowed to gelate at 37 °C for 30 min. Subsequently, LEPCs (1 × 10^7^/mL) were seeded on the hydrogel and incubated for 2 h. The hydrogel containing the cells was fixed in 5% glutaraldehyde. After gradient ethanol dehydration, the specimen was dried in vacuum and coated with platinum. The nanofibrous scaffold formed by SAP and spreading of the cells on the scaffold were examined with a scanning electron microscope (Su8010, Hitachi, Tokyo, Japan).

### Assessment of cytoprotective effect of SAP

To imitate hypoxic and ischemic microenvironment of the infarcted myocardium, the cells in SAP hydrogel were treated with hypoxia (1% O_2_) and low serum (2% FBS). After treatment for 2 h, the cells were incubated with cell counting kit-8 (CCK-8; Dojindo, Kumamoto, Japan), and the absorbance at OD_450_ was measured using a microplate reader (Tecan Infinite 200, Mannedort, Switzerland). Moreover, the apoptotic and necrotic cells in the treated cells were examined with ethidium bromide and acridine orange (EB/AO) staining. The experiment was repeated for six times.

For assessing cytoprotective effect of SAP in the ischemic tissue, implantation of LEPCs into abdominal pouches was performed as previously described [50]. Briefly, SAP solution containing Dil-labeled cells was added on the poriferous polyethylene terephthalate membranes removed from the transwells (Becton–Dickinson, Franklin Lakes, NJ, USA) and incubated for 2 h. After dissecting the skin of the anterior abdominal wall at the median line, the superficial fascia at both sides was bluntly dissected with a forceps to create two pouches, and the vessels of the pouches were ligated. Then, the cell-loaded membranes were implanted into the pouches; cell side of the membrane was towards abdominal muscles. At 24 h after implantation, the membranes were taken out gently and the survived cells (rest DiI-labelled cells) were counted with a fluorescent microscope.

### Detection of VEGF-C released from SAP hydrogel

VEGF-C (10 μg/mL) was mixed with the SAP solution (10 μL, 1:1). The mixture was dropped into 96-well plate and allowed to gelate at 37 °C for 30 min. After adding 200 μL 0.01 M PBS, the hydrogel was incubated for 28 days. At 1 day, 3 days, 7 days, 14 days and 28 days after incubation, the supernatant was pipetted and then replaced with the same amount of PBS. Concentration of VEGF-C released into the supernatant was measured by enzyme-linked immunosorbent assay (ELISA). The cumulative released VEGF-C over time was analyzed using GraphPad Prism 5. The experiment was repeated for six times.

### Assay of cell migration from SAP hydrogel

SAP solution (5 μL) containg 1 × 10^4^ cells and 10 ng VEGF-C was added in a 24-well plate and incubated at 37 °C for 30 min to be allowed to gelate. Then, 200 μL medium supplemented with 15% FBS was added and continued to be incubated. The migrated cells from SAP hydrogel were examined using a phase contrast microscope.

### Cell transplantation

Rat MI models were established by ligation of the left anterior descending (LAD) coronary artery according to the procedure described previously [48]. 74 male SD rats (8‒10 w, 200‒250 g) were anesthetized with intraperitoneal injection of ketamine (80 mg/kg) and xylazine (5‒10 mg/kg). In sham group (*n* = 7), the needle passed through the ventricular wall around LAD artery, but was not ligated. LAD artery was permanently ligated in 67 rats. At day 7 post-MI, 3 rats died of heart failure. 2 rats were kicked out because MI is not obvious. The rest of the rats was divided into control (PBS, *n* = 7), LEPCs (1 × 10^6^ cells, *n* = 7), VEGF-C (1 μg, *n* = 7), SAP (0.1 mg, *n* = 10), SAP + LEPCs (*n* = 10), SAP + VEGF-C (*n* = 10), SAP + LEPCs + VEGF-C (*n* = 11) groups. After anesthetization, the rats were intubated endotracheally and connected to an animal ventilator (HX101-E, Techman, Chengdu, Sichuan, China). The anterior thoracic wall was incised at the left third intercostal space and distracted with a sternum spreader. Intramyocardial injection (80 μL in volume) was performed at 4 points of the peri-infarct region. Then, the thoracic wall was stitched layer by layer.

### Echocardiography

Echocardiograms were recorded with an echocardiograph (Visual Sonics, Toronto, Canada) before MI, at 1 week after MI (before transplantation) and at 4 weeks after transplantation. The M-mode cursor was positioned to the parasternal long axis and viewed at the level of papillary muscles. The echocardiograms were measured to obtain the left ventricular end-diastolic diameter (LVEDD), left ventricular end-systolic diameter (LVESD), left ventricular end-diastolic volume (LVEDV) and left ventricular end-systolic volume (LVESV) from at least three consecutive cardiac cycles. To evaluate cardiac function, the ejection fraction (EF) and the fractional shortening (FS) were calculated by the following formulae: EF (%) = (LVEDV − LVESV)/LVEDV × 100, FS (%) = (LVEDD − LVESD)/LVEDD × 100. Successful establishment of MI model was confirmed by EF of < 50% and FS of < 30%.

### Assessment of VEGF-C and VEGF paracrine

After treatment with hypoxia (1% O_2_) for 2 h, VEGF-C and VEGF secreted from the cells in SAP were detected with VEGF-C and VEGF ELISA kits (Guchen, Shanghai, China). Absorbance was measured with a microplate reader (Tecan Infinite 200, Mannedorf, Switzerland) at 450 nm. Moreover, VEGF-C and VEGF in the plasma and myocardium of the peri-infarct region were detected at 4 weeks after transplantation. The mass of the myocardium was weighed and homogenized in 0.01 M PBS at a ratio of 1 g tissue to 9 mL PBS. The homogenate was sonicated with an ultrasonic cell disrupter (Branson, Danbury, CT, USA) and centrifuged for 5 min at 5000 g to get the supernatant.

### Gravimetry

Cardiac water content was evaluated with wet weight–dry weight method similar to described previously [26]. Establishment of rat MI models and division of the rats were same as above; six rats were used for each group. Tissue samples were harvested from the infarct region, peri-infarct region, interventricular septum and whole left ventricle at 4 weeks after transplantation. The wet weight of each sample was measured with an electronic balance (Shuangquan, Shanghai, China). The dry weight was recorded after desiccating samples for 5 days at 70 °C. Cardiac water content was calculated by the following formula: water content (%) = (wet weight − dry weight)/wet weight × 100.

### Histologic examination

At 4 weeks after transplantation, the hearts were harvested and fixed with paraformaldehyde over night. After gradually dehydrated with 15% and 30% sucrose, the hearts were embedded with Tissue-Tek OCT (Sakura Finetek, Torrance, CA, USA). The cryostat sections of 5-µm thickness were obtained from the upper, middle and lower parts of the infarct region and stained with Masson’s trichrome. The area of scar was calculated as percentage of the circumference of the infarct region in the circumference of the whole left ventricular wall by ImageJ 1.46r (Wayne Rasband, NIH, USA). The thickness of the ventricular wall at the infarct region was measured at the thinnest part of the region.

For assessing compatibility of the designer SAP with the transplanted cells and myocardium, the suspension of 1 × 10^5^ cells in 10 μL SAP solution was intramyocardially injected into the peri-infarct region. At 6 h and 4 weeks after transplantation, the myocardium at injection site was removed and fixed in 5% glutaraldehyde, respectively. Following dehydration with alcohol and acetone, the tissue block was embedded in resin. Ultrathin sections were prepared and stained with uranyl acetate and lead citrate. Distribution of SAP nanofibers carrying LEPCs in the myocardium was examined with a transmission electron microscope (FEI, Hillsboro, OR, USA).

### Immunostaining

To evaluate myocardial repair and improvement of inflammation after transplantation, the infarct region was examined with cTnT and Cx43 immunostaining and CD68 immunostaining, respectively. Lymphangiogenesis and angiogenesis of the peri-infarct region were assessed with LYVE-1 and CD31 immunostaining, respectively. At least 5 fields (200 ×) were randomly selected in each section. Densities of the lymphatic vessels and microvessels were assessed by measuring the area of the transverse sections of LYVE-1^+^ vessels with ImageJ 1.46r software (Wayne Rasband, NIH, USA) and counting CD31^+^ vessels, respectively, from at least 3 independent frozen sections. To trace the engrafted cells, the cells were transfected with GFP-lentivirus (Sangon Biotech, Shanghai, China) at a MOI of 30 for 72 h before transplantation. Differentiation of the engrafted cells into LECs was determined with GFP and LYVE-1 double staining. The antibodies used for immunostaining are described in the Supplemental Table 1.

### Whole-mount staining

For objectively evaluating cardiac lymphangiogenesis after transplantation, the subepicardial lymphatic vessels at the peri-infarct and infarct regions were demonstrated with whole-mount staining. The hearts were fixed for 2 h in 4% paraformaldehyde, and rinsed for 10 min in PBS for 3 times. Then, the hearts were treated with 1% Triton X-100 for 1 h, and incubated in 10% serum for 30 min. Subsequently, the hearts were incubated with rabbit anti-LYVE-1 antibody (1:1000, Novus Biologics) for 2 h and with Alexa Fluor 488 conjugated goat anti-rabbit IgG (1:1000; Jackson) for 1 h. The distribution of the subepicardial lymphatic vessels was photographed with a fluorescence microscope (Olympus, Tokyo, Japan). The images were processed with ImageJ 1.46r software.

### Statistical analysis

Statistical analyses were performed with GraphPad Prism 5 (GraphPad Software, La Jolla, CA, USA). All data were evaluated with the Gaussian distribution by Kolmogorov–Smirnov test and presented as mean values ± standard deviations. Statistical differences between groups were evaluated by Student’s *t* test or one-way analysis of variance (ANOVA) followed by Tukey post hoc test, or two-way ANOVA followed by Bonferroni post test for samples with normal distribution. For samples with non-Gaussian distribution, nonparametric Kruskal–Wallis followed by Dunns post hoc test was used. Correlation was evaluated by Pearson test. A *p* value of < 0.05 was considered as statistically significant.

## Results

### Differentiation of CD34^+^VEGFR-3^+^ cells and their participation in Lymphangiogenesis

**Fig. 1:**
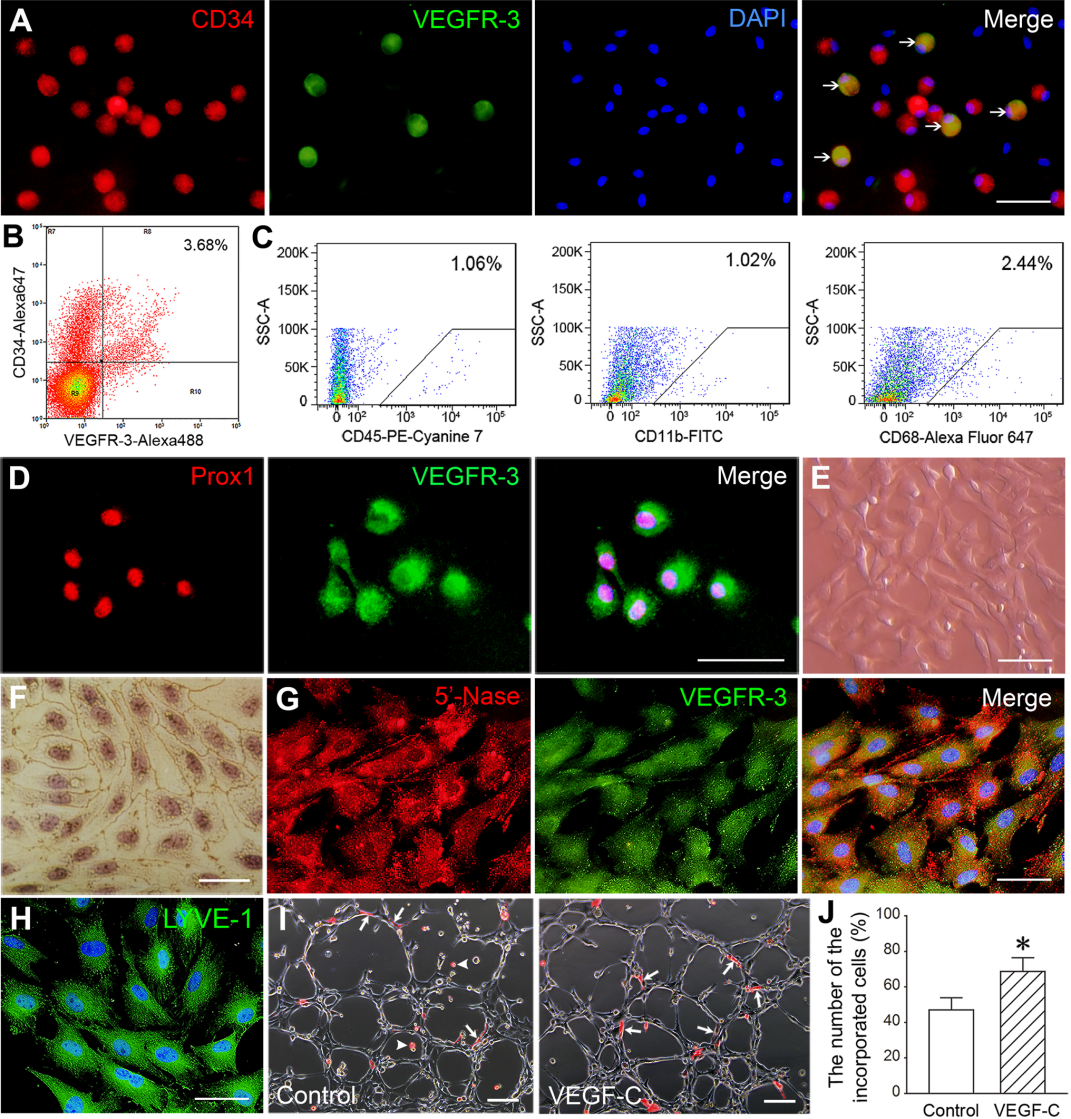
Characterization of CD34^+^VEGFR-3^+^ cells. **a** CD34^+^VEGFR-3^+^ cells (arrows) in the mononuclear cells isolated from rat bone marrow. **b** The phenotype of the mononuclear cells analyzed by dual-color flow cytometry. Percentage of the positive cells was compared to isotype control. **c** The flow cytometric analysis of CD45, CD11b and CD68 in the sorted CD34^+^VEGFR-3^+^ cells. **d** The sorted CD34^+^VEGFR-3^+^ cells expressed Prox1. **e** The phase contrast image of the sort cells at day 14 after induction with VEGF-C. **f** The endothelium-like feature of the monolayer of the differentiated cells. Silver nitrate staining. **g**, **h** The differentiated cells expressed 5′-Nase, VEGFR-3 and LYVE-1. **i** CD34^+^VEGFR-3^+^ LEPCs incorporated into the capillary-like structures formed by the endothelial cells isolated from rat thoracic duct. LEPCs were labeled with Dil. Arrowheads indicate free LEPCs. **j** The statistical result of the number of the incorporated cells. *p < 0.01 versus control group. *n* = 6. Scale bar = 100 μm (**e**, **i**) and 50 μm (**a**, **d**, **f**, **g**, **h**)

There was a population of CD34^+^VEGFR-3^+^ cells in the mononuclear cells isolated from rat bone marrow (Fig. 1a). The result of FACS reveals that CD34^+^VEGFR-3^+^ cells are 3.68% in the mononuclear cells (Fig. 1b). The sorted CD34^+^VEGFR-3^+^ cells were negative for hematopoietic marker (CD45) and monocyte–macrophage markers (CD11b and CD68) and positive for Prox1 (Fig. 1c, d). At day 14 after induction with VEGF-C, the cells displayed fusiform or polygonal shape; the confluent monolayer of the cells demonstrated a typical cobblestone appearance like endothelial cells and was positive for silver nitrate staining (Fig. 1e, f). The differentiated cells expressed lymphatic endothelial cell makers 5′-Nase, VEGFR-3 and LYVE-1 (Fig. 1g, h). In three-dimensional Matrigel, CD34^+^VEGFR-3^+^ cells could incorporate into the lymphatic capillary-like structures formed by LECs. Compared with control group, the number of the incorporated cells in VEGF-C group was greater (Fig. 1i, j).

### Cytoprotective effect of SAP

**Fig. 2:**
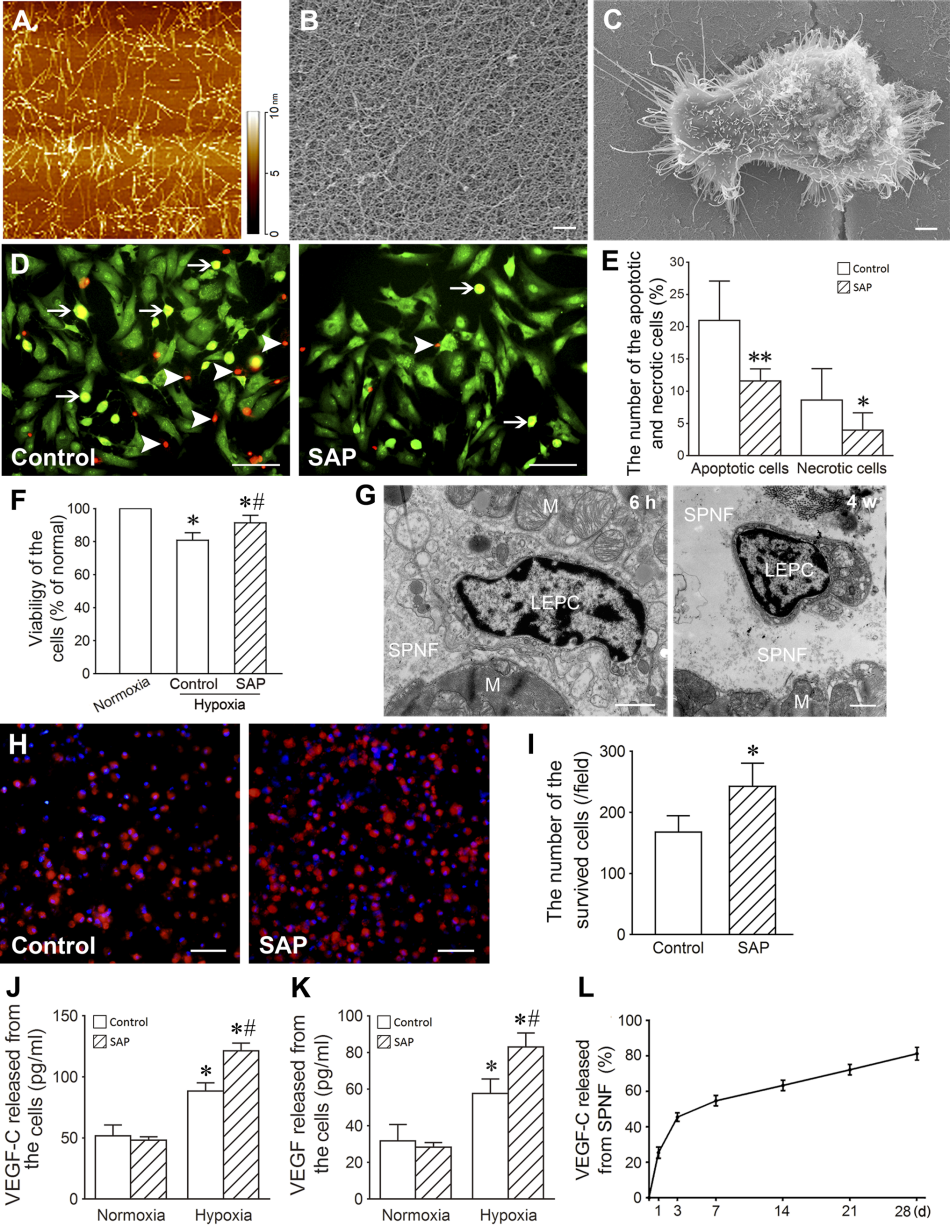
Biological properties of SAP. **a** The nanofibers formed by SAP. Atomic force microscopic image. **b** The scaffolds organized by SAP nanofibers. Scanning electron microscopic image. Scale bar = 500 nm. **c** A cell spreading on SAP nanofibers. Scanning electron microscopic image. Scale bar = 2 μm. **d** The apoptotic (arrows) and necrotic (arrowheads) cells in the cells treated with hypoxia and serum deprivation for 2 h. EB/AO staining. Scale bar = 100 μm. **e** The statistical result of the apoptotic and necrotic cells. *p < 0.05, **p < 0.001 versus control group. *n* = 6. **f** Viability of the cells treated with hypoxia and serum deprivation for 2 h. CCK-8 assay. *p < 0.001 versus normoxia group, ^#^*p *< 0.001 versus control group. *n* = 6. **g** The SAP nanofibers (SAPN) and cells (LEPC) in the myocardium (M) at 6 h and 4 weeks after transplantation. Transmission electron microscopic image. Scale bar = 1 μm. **h** The survived cells after transplantation into the ischemic abdominal pouch for 24 h. The cells were labeled with Dil. Scale bar = 100 μm. **i** The statistical result of the survived cells in the ischemic abdominal pouch. *p < 0.01 versus control group. **j**, **k** Concentration of VEGF-C and VEGF in the supernate of the cells. *p < 0.01 versus normoxia group, ^#^*p *< 0.01 versus control group. *n* = 6. **l** The curve of the sustained release of VEGF-C from SAP hydrogel. *n* = 6

The molecular weight of the fabricated SAP was 2128.17; its purity was 95.27%. At 30 min after sonication, SAP formed spontaneously nanofibers with diameter of 5–10 nm and length of 20–200 nm (Fig. 2a). At 2 h after sonication, the nanofibers further assembled into scaffolds with apertures of 20–400 nm (Fig. 2b). LEPCs adhered and spread well on the SAP nanofibers (Fig. 2c). In the conditions of hypoxia and serum deprivation, the numbers of the apoptotic and necrotic cells in SAP group were lesser than control group (Fig. 2d, e). Moreover, viability of the cells in SAP group was higher (Fig. 2f). After transplantation, SAP nanofibers were located between the bundles of the myocardium and exhibited good compatibility with the transplanted cells and surrounding myocardium. At 4 weeks after transplantation, SAP nanofibers were partly degraded (Fig. 2g). At 24 h after transplantation into the ischemic abdominal pouches, the survived cells in SAP group were more than in control group significantly (Fig. 2h, i). The experimental data show that SAP protects the cells to survive. After treatment with hypoxia for 2 h, concentration of VEGF-C and VEGF in the supernatant of the cells carried with SAP hydrogel was increased (Fig. 2j, k).

### The sustained release of VEGF-C from SAP hydrogel

Concentration of VEGF-C in the supernatant represented an initial rapid release by day 3, followed by a steady and lasting release over 28 days from SAP hydrogel. At day 28 after incubation, the cumulated volume of VEGF-C released from SAP hydrogel was 83.48 ± 2.102% (Fig. 2l).

### Migration of the cells from SAP hytrogel

Compared with the control group, the number of the migrated cells from SAP hytrogel containing VEGF-C was greater (Supplemental Fig. 1).

### Improvement of the cardiac function after transplantation

**Fig. 3:**
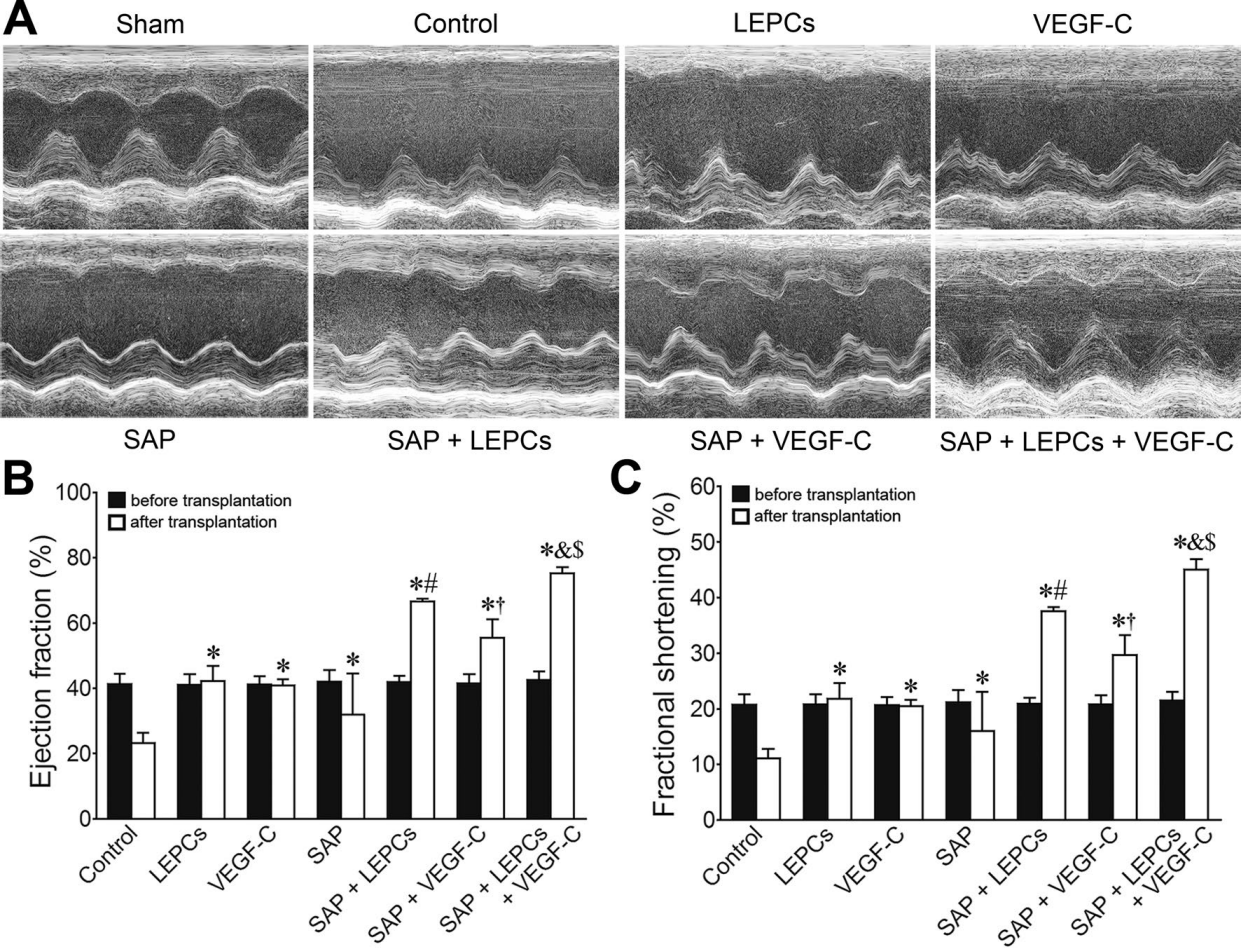
Changes of cardiac function after transplantation. **a** Representative echocardiograms of the free wall of the left ventricle at 4 weeks after transplantation. Contraction of the left ventricle in SAP + LEPCs + VEGF-C group was improved significantly. **b**, **c** Statistical results of EF and FS. *p < 0.001 versus control group, ^#^*p *< 0.001 versus LEPCs group, ^†^*p *< 0.001 versus VEGF-C group, ^&^*p *< 0.001 versus SAP + LEPCs group, ^$^*p *< 0.001 versus SAP + VEGF-C group

Echocardiography revealed that the cardiac function in all rats with occlusion of LAD artery was severely compromised at 1 week after ligation of LAD artery. In sham group, the heart exhibited regular systole and diastole; EF and FS were 85.46 ± 2.949 and 55.64 ± 3.559, respectively. In control group, the cardiac cavity was dilated, the anterior wall of the left ventricle was thinned, and the systolic wave of the anterior wall was weak post-MI. Loss of the cardiac function lasted for following 4 weeks. At 4 weeks after transplantation, the function of the hearts implemented LEPCs; VEGF-C or SAP transplantation was significantly improved. EF and FS in SAP + LEPCs and SAP + VEGF-C groups were greater than that in LEPCs or VEGF-C group, respectively. Compared with SAP + LEPCs or SAP + VEGF-C groups, EF and FS in SAP + LEPCs + VEGF-C group were increased significantly (Fig. 3).

### Changes of cardiac edema after transplantation

**Fig. 4:**
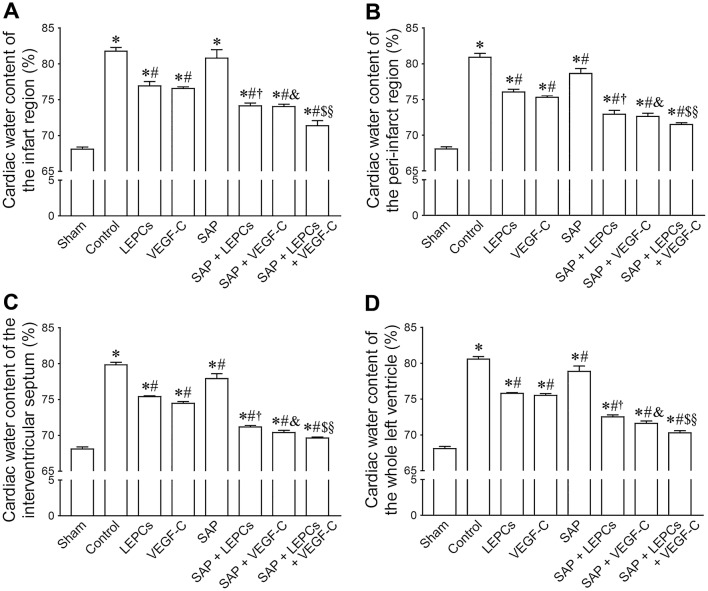
Water content of the infarct region (**a**), peri-infarct region (**b**), interventricular septum (**c**) and whole left ventricle (**d**). *p < 0.01 versus sham group, ^#^*p *< 0.05 versus control group, ^†^*p *< 0.01 versus LEPCs group, ^&^*p *< 0.01 versus VEGF-C group, ^$^*p *< 0.05 versus SAP + LEPCs group, ^§^*p *< 0.05 versus SAP + VEGF-C group. *n* = 6

Cardiac water content soared post-MI, especially in the center of the infarct region. At 4 weeks after transplantation, water content of the infarct region, peri-infarct region, interventricular septum and whole left ventricle was measured. In control group, water content increased dramatically. Except the infarct region, water content of the above sites in LEPCs, VEGF-C and SAP groups was lesser than that in control group. Compared with LEPCs or VEGF-C group, water content in SAP + LEPCs and SAP + VEGF-C groups decreased, respectively. Reduction of water content in SAP + LEPCs + VEGF-C group was significant (Fig. 4).

### Morphological changes of the left ventricle after transplantation

**Fig. 5:**
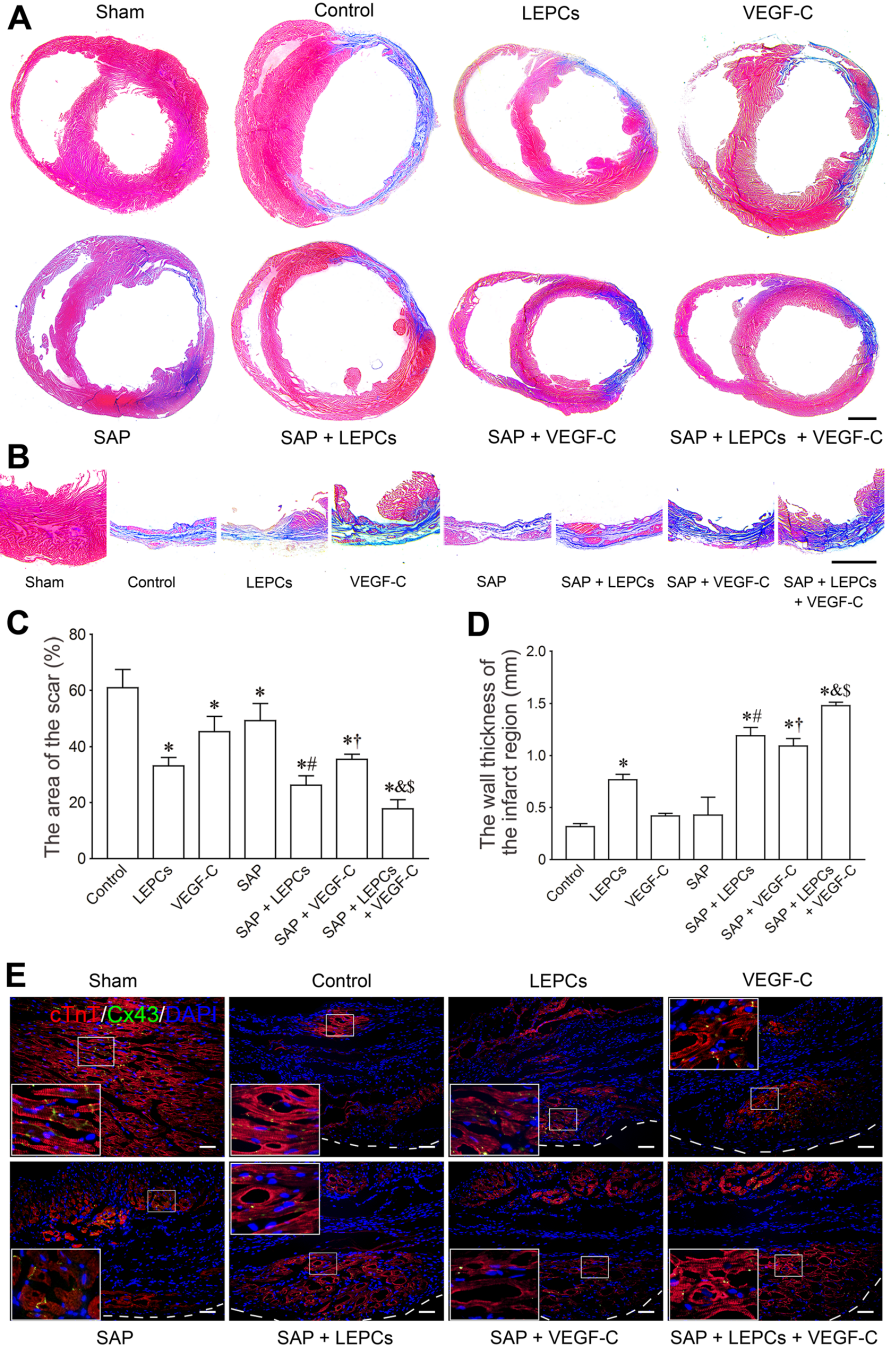
Histological changes of the free wall of the left ventricle after transplantation. **a** The transverse sections of the ventricles at the widest part of the infarct region. Masson’s trichrome. Scale bar = 2 mm. **b** The transverse sections at the thinnest part of the infarct region. Masson’s trichrome staining. Scale bar = 2 mm. **c**, **d** The statistical results of the area of the scar and thickness of left ventricular wall at the thinnest part of the infarct region. *p < 0.001 versus control group, ^#^*p *< 0.05 versus LEPCs group, ^†^*p *< 0.01 versus VEGF-C group, ^&^*p *< 0.05 versus SAP + LEPCs group, ^$^*p *< 0.05 versus SAP + VEGF-C group. **e** Myocardial regeneration at the infarct region. The white dash line indicates the surface of the epicardium. The large box is magnification of the small box. cTnT and Cx43 immunostaining. Scale bar = 100 μm

At 4 weeks post-MI, the myocardial tissue in the scarred region was replaced by fibrous tissue in control group. The free wall of the left ventricle was thinned. The cavity of the left ventricle was dilated obviously. After transplantation, there was more myocardial tissue at the infarct region in LEPCs, SAP + LEPCs, SAP + VEGF-C, SAP + LEPCs + VEGF-C groups, especially in the latter (Fig. 5a, b). The size of the scar in LEPCs group was lesser than that in control group. Compared with LEPCs and VEGF-C groups, the scar in SAP + LEPCs and SAP + VEGF-C groups was smaller, respectively. In the SAP + LEPCs + VEGF-C group, the scar was decreased greatly (Fig. 5c). The changes in the thickness of left ventricular wall are shown in Fig. 5d. There was more myocardium at the infarct region in SAP + LEPCs, SAP + VEGF-C, and SAP + LEPCs + VEGF-C groups (Fig. 5e).

### Survival and differentiation of the engrafted cells

**Fig. 6:**
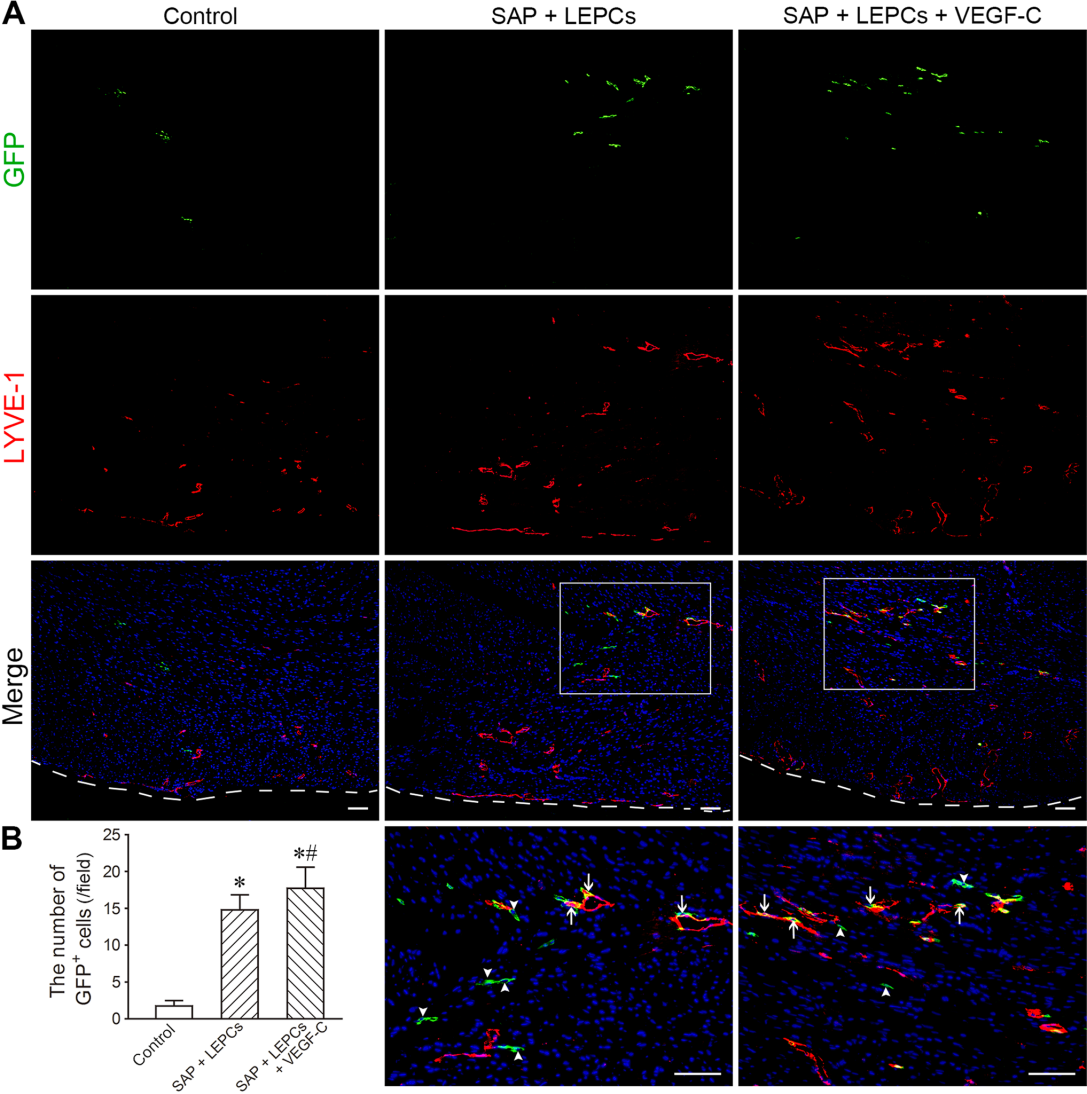
The survived and differentiated cells in the engrafted cells. **a** The distribution of GFP^+^ cells in infarct border zone. The white dash line indicates the surface of the epicardium. The panels of the fourth line are magnification of the boxes in the panels of the third line. Arrowheads and arrows indicate GFP^+^ cells and the differentiated cells, respectively. Scale bar = 100 μm. **b** The statistical result of the number of GFP^+^ cells. *p < 0.001 versus control group

The survival of the engrafted cells was determined by the remanent GFP^+^ cells at the peri-infarct region at 4 weeks after transplantation. The number of GFP^+^ cells in SAP + LEPCs or SAP + LEPCs + VEGF-C group was greater than that in control group (LEPCs only) significantly (Fig. 6a, b). Double staining of GFP and LYVE-1 showed that some GFP^+^ cells expressed LYVE-1 and incorporated into lymphatic vessels. There were more differentiated and incorporated cells in SAP + LEPCs + VEGF-C group than that in SAP + LEPCs group (Fig. 6a). None of the transplanted CD34^+^VEGFR-3^+^ EPCs was observed to differentiate into vascular endothelial cells (data not shown).

### Changes of VEGF-C and VEGF concentration in plasma and myocardium

At 4 weeks after transplantation, the concentration of VEGF-C and VEGF in plasma and myocardium of the peri-infarct region was high in LEPCs or VEGF-C groups than that in control group. Compared with LEPCs and VEGF-C groups, the concentration of VEGF-C and VEGF in SAP + LEPCs and SAP + VEGF-C groups was increased, respectively. The concentration of VEGF-C and VEGF in SAP + LEPCs + VEGF-C group was high significantly (Supplemental Fig. 2). With paracrine of the cells in vitro, these results in vivo indicate that the sustained release of the SAP hydrogel and paracrine of LEPCs account for increase of VEGF-C and VEGF concentration.

### Lymphangiogenesis and angiogenesis after transplantation

**Fig. 7:**
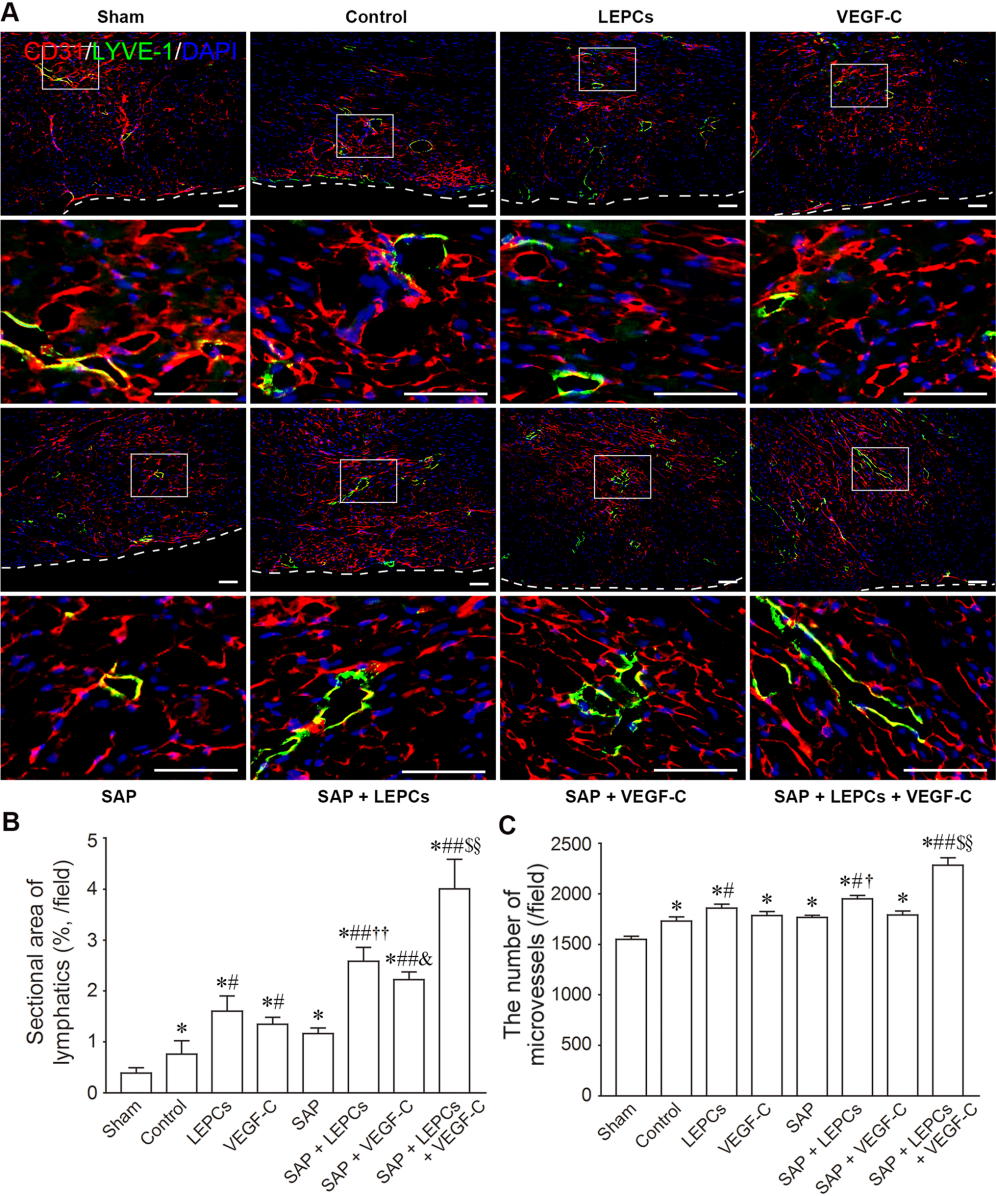
The lymphatic vessels and microvessels at the peri-infarct region at 4 weeks after transplantation. **a** The distribution of the lymphatic vessels and microvessels. LYVE-1 and CD31 immunostaining. The panels of the second and fourth lines are magnification of the boxes in the panels of the first and third lines, respectively. Scale bar = 100 μm. **b** The area of transverse section of LYVE-1^+^ lymphatic vessels. **c** The number of CD31^+^ microvessels. *p < 0.001 versus sham group, ^#^*p *< 0.01 and ^##^*p *< 0.001 versus control group, ^†^*p *< 0.05 and ^††^*p *< 0.001 versus LEPCs group, ^&^*p *< 0.001 versus VEGF-C group, ^$^*p *< 0.001 versus SAP + LEPCs group, ^§^*p *< 0.001 versus SAP + VEGF-C group

The lymphatic vessels and microvessels at the peri-infarct region were demonstrated with LYVE-1 and CD31 immunostaining, respectively (Supplemental Fig. 3, Fig. 7a). The area of the transverse sections of the lymphatic vessels in SAP + LEPCs and SAP + VEGF-C groups was more than that in LEPCs or VEGF-C alone group, respectively. Compared with SAP + LEPCs or SAP + VEGF-C groups, the area of the transverse sections of the lymphatic vessels in SAP + LEPCs + VEGF-C group was greater (Fig. 7b). The microvessels in SAP + LEPCs group were increased. Difference in the number of the microvessels between SAP + LEPCs + VEGF-C group and SAP + LEPCs group was significant (Fig. 7c). The distribution of the subepicardial lymphatic vessels was examined with whole-mount staining. There were less lymphatic vessels at the infarct regions in control group than sham group. In SAP + LEPCs + VEGF-C group, the lymphatic vessels were increased dramatically (Supplemental Fig. 4). These results demonstrate that delivery of LEPCs and VEGF-C with SAP enhances cardiac lymphangiogenesis.

### Clearance of the inflammatory cells in the infarct region

**Fig. 8:**
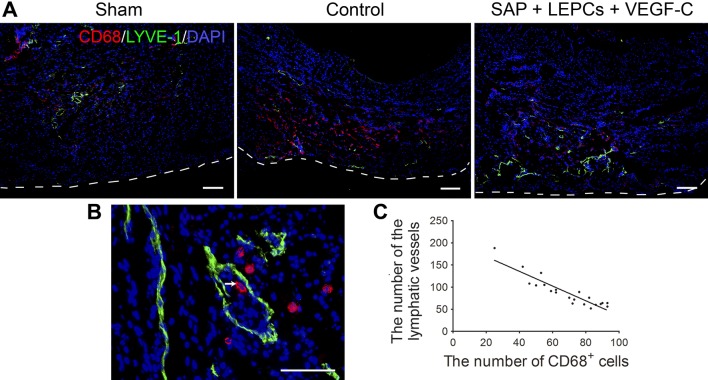
The lymphatic vessels and CD68^+^ cells in the infarct region at 4 weeks after transplantation. **a** The distribution of the lymphatic vessels and CD68^+^ cells. The white dash line indicates the surface of the epicardium. Immunostaining. Scale bar = 200 μm. **b** A lymphatic vessel contains a CD68^+^ cells (arrows). Scale bar = 100 μm. **c** Correlation of LYVE-1^+^ lymphatic vessels and CD68^+^ cells. Slope = − 1.66, *r*^2^ = 0.8374, *p *< 0.0001

Relation of density of lymphatic vessels with transportation of the inflammatory cells was analyzed with double staining of LYVE-1 and CD68. At 4 weeks post MI, there were numerous CD68^+^ inflammatory cells in control group. In SAP + LEPCs + VEGF-C group, the lymphatic vessels were increased, while CD68^+^ cells were decreased markedly (Fig. 8a). Some lymphatic vessels contained CD68^+^ cells (Fig. 8b). There was a linear correlation between the numbers of lymphatic vessels and CD68^+^ cells (Fig. 8c).

## Discussion

In this study, we demonstrate that combination of LEPC transplantation and VEGF-C release with SAP is effective for enhancing cardiac lymphangiogenesis, reducing cardiac edema and inflammation, attenuating reverse myocardial remodeling and improving cardiac function post-MI. Our findings provide the first evidence for cardiac lymphangiogenesis participated by bone marrow-derived LEPCs. CD34^+^VEGFR-3^+^ EPCs isolated from the mononuclear cells of bone marrow express Prox1 and have a potential to differentiate towards lymphatic endothelial cells. Furthermore, the cells can incorporate into the lymphatic vessels in the ischemic myocardium after transplantation. Another study suggests that bone marrow-derived podoplanin^+^ cells may participate in lymphatic neovascularization under inflammatory and tumorigenic conditions [21]. Our observations, together with other studies [18, 21, 34, 40], suggest that the processes of postnatal LEPC-induced lymphangiogenesis include mobilization from bone marrow to peripheral blood, transmigration through blood capillaries, homing to the local tissue, differentiation into lymphatic endothelial cells and participation in lymphatic neovascularization. However, effects of the endogenous LEPCs on lymphangiogenesis in MI need further investigation. In recent years, more attention has been paid on paracrine mechanisms of reparative and regenerative effects of stem cells [13]. Besides differentiation towards lymphatic endothelial cells, VEGF-C production accounts for lymphangiogenic effect of the implanted LEPCs. In addition, this study shows that VEGF produced by the cells may stimulate angiogenesis in the infarcted myocardium. Therefore, we suggest that CD34^+^VEGFR-3^+^ LEPCs isolated from bone marrow represent a novel source of the seed cells for cell-based MI therapies. Moreover, our experimental data reveal that myocardial regeneration is enhanced after transplantation of LEPCs and VEGF-C loaded with SAP hydrogel. Although the mechanisms of myocardial regeneration are complex, improvement of microenvironment after transplantation might be beneficial for differentiation of epicardium-derived cells towards cardiomyocytes [38] and cardiomyocyte division [43]. Myocardial contraction is the major determinant of myocardial lymph drainage [4]. Myocardial lymphostasis during cardioplegic arrest may contribute to postoperative myocardial edema and left ventricular dysfunction [28]. Cardiac arrhythmia is associated with lymphedema, probably due to irregular contractions impeding lymphatic drainage [31]. Therefore, simultaneous transplantation of the stem cells which differentiate towards cardiomyocytes such as mesenchymal stem cells may increase efficiency of improving lymphatic drainage.

The results of this study show that VEGF-C released from SAP promotes cardiac lymphangiogenesis via inducting differentiation of the transplanted LEPCs and stimulating growth of the pre-existed lymphatic vessels. Moreover, VEGF-C promotes migration of LEPCs from SAP. Both the transplanted cells and cardiac lymphatic vessels express VEGFR-3. In adult, VEGFR-3 expression is largely restricted to lymphatic endothelium [1]. Expression of VEGFR-3 in the myocardium increases significantly post-MI [12, 20]. The ligands of VEGFR-3 are VEGF-C and VEGF-D. Inhibition of VEGF-C/VEGFR-3 signaling pathway suppresses cardiac lymphangiogenesis [37]. Moreover, silencing expression of VEGFR-3 mRNA inhibits lymphangiogenesis of LEPCs [22]. This study highlights the pivotal roles of VEGF-C/VEGFR-3 signaling in cardiac lymphangiogenesis. Our experimental data demonstrate that LEPC transplantation and SAP-released VEGF-C promote cardiac angiogenesis. VEGF-C may act on VEGFR-2 on vascular endothelial cells [16], activating cardiac angiogenesis [24, 47]. Therefore, VEGF secreted from the transplanted cells or VEGF-C released from SAP can induce growth of the microvessels. On the other hand, VEGF-C may also increase vascular permeability [5]. In this study, mild effect of VEGF-C sustainedly released from SAP is beneficial to avoiding aggravation of blood vascular hyperpermeability.

Our experimental data indicate that the RADA16-I peptide modified with adhesion motif RGDS can retain LEPCs in the transplanted site effectively through specific RGDS-integrin binding. In the condition of hypoxia and ischemia, the SAP hydrogel exerts cytoprotective effect. SAP represents good biocompatibility and safe biodegradability without immunogenicity, and mimics the natural cardiac extracellular matrix [33]. In the infarcted myocardium, the extracellular matrix is degraded so that ventricular wall is weakened and thinned [8]. Intramyocardial injection of SAP prevents ventricular remodeling [23]. SAP creates nanofiber microenvironment in the myocardium and promotes vascular cell recruitment and vascularization [7]. Stem cell-based therapies have been limited by poor survival and differentiation of the engrafted cells within ischemic and inflammatory microenvironment [3]. SAP favors survival of the transplanted cells [7]. Delivery of bone marrow-derived mononuclear cells with SAP improves cell retention and cardiac function post-MI [23]. Transplantation of cardiac progenitor cells carried with RADA16-I peptide prevents cardiac remodeling and dysfunction [41]. This study reveals that intramyocardial delivery with the SAP hydrogel promotes survival and differentiation of the transplanted LEPCs. Moreover, the SAP absorbs and encapsulates VEGF-C, avoiding its repaid washing into the bloodstream and degradation. Delivery of VEGF-C with albumin–alginate microparticles, gelatin hydrogel or fibrin augments lymphangiogenesis in the infarcted myocardium [12, 37] and wound tissue [11], respectively. Following degradation of SAP, SAP-carried growth factor is released sustainedly [10]. Therefore, the SAP is reliable in the sustained release of VEGF-C. The dramatical increase of the myocardial VEGF-C level at 4 weeks after transplantation is associated with protection of SAP against VEGF-C degradation as well as paracrine of the implanted LEPCs. Moreover, secretion of the regenerated cardiac cells or the cells recruited by VEGF-C might be involved in the increase of VEGF-C level. The prolonged higher VEGF-C level is effective for stimulating cardiac lymphangiogenesis.

In summary, this study demonstrates that the combined delivery of CD34^+^VEGFR-3^+^ LEPCs and VEGF-C with the functionalized SAP is potent for stimulating cardiac lymphangiogenesis. The SAP hydrogel is a novel vehicle for delivery of LEPCs and VEGF-C. Besides differentiation towards lymphatic endothelial cells, paracrine mechanism of CD34^+^VEGFR-3^+^ LEPCs is involved in lymphangiogenesis and angiogenesis. VEGF-C sustainedly released from SAP has effects on differentiation of LEPCs into endothelial cells and growth of pre-existing lymphatic vessels. Improvement of the edematous and inflammatory microenvironment after transplantation enhances angiogenesis and myocardial regeneration, resulting in reduction of reverse remodeling and improvement of cardiac function. Therefore, targeting cardiac lymphangiogenesis using LEPC transplantation and VEGF-C release from SAP is a feasible strategy for therapeutic intervention of cardiac edema in cardiovascular diseases.

## Electronic supplementary material

Below is the link to the electronic supplementary material.

Supplementary material 1 (DOCX 1246 kb)
