# Supplementary material for: Enhancement of cardiac lymphangiogenesis by transplantation of CD34+VEGFR-3+ endothelial progenitor cells and sustained release of VEGF-C
Source: Basic Res Cardiol. 2019 Oct 6;114(6):43. doi: 10.1007/s00395-019-0752-z (PMC6778587; doi:10.1007/s00395-019-0752-z)
Supplement: Supplementary file 1 — Supplementary material 1 (DOCX 1246 kb) [file 395_2019_752_MOESM1_ESM.docx]

**Electronic Supplementary Material**

**Supplemental Table 1.**

**Table S1 The antibodies used for immunostaining**

| Antibodies | Dilution | Company |
| --- | --- | --- |
| Chicken anti-GFP | 1:100 | Novus Biologics, Littleton, CO, USA |
| Goat anti-chicken (conjugated with DyLight 488) | 1:400 | Novus Biologics, Littleton, CO, USA |
| Goat anti-mouse (conjugated with Alexa Fluor 594) | 1:400 | Jackson, West Grove, PA, USA |
| Goat anti-mouse (conjugated with Alexa Fluor 647) | 1:400 | Jackson, West Grove, PA, USA |
| Goat anti-rabbit (conjugated with Alexa Fluor 488) | 1:400 | Jackson, West Grove, PA, USA |
| Goat anti-rabbit (conjugated with DyLight 594) | 1:400 | Abcam, Cambridge, MA, USA |
| Mouse anti-5’-Nase | 1:200 | From Dr. Seiji Kato, Japan |
| Mouse anti-CD31 | 1:200 | Abcam, Cambridge, MA, USA |
| Mouse anti-CD34 | 1:200 | Santa Cruz, CA, USA |
| Mouse anti-CD45 (conjugated with PE-Cyanine 7) | 1:100 | eBioscience, San Diego, CA, USA |
| Mouse anti-CD68 | 1:100 | Santa Cruz, CA, USA |
| Mouse anti-cTnT | 1:200 | Santa Cruz, CA, USA |
| Mouse anti-Prox1 | 1:100 | Abcam, Cambridge, MA, USA |
| Rabbit anti-CD11b (conjugated with FITC) | 1:100 | eBioscience, San Diego, CA, USA |
| Rabbit anti-Cx43 | 1:200 | Santa Cruz, CA, USA |
| Rabbit anti-LYVE-1 | 1:200 | Novus Biologics, Littleton, CO, USA |
| Rabbit anti-VEGFR-3 | 1:200 | Santa Cruz, CA, USA |

**Supplemental Fig. 1**

**
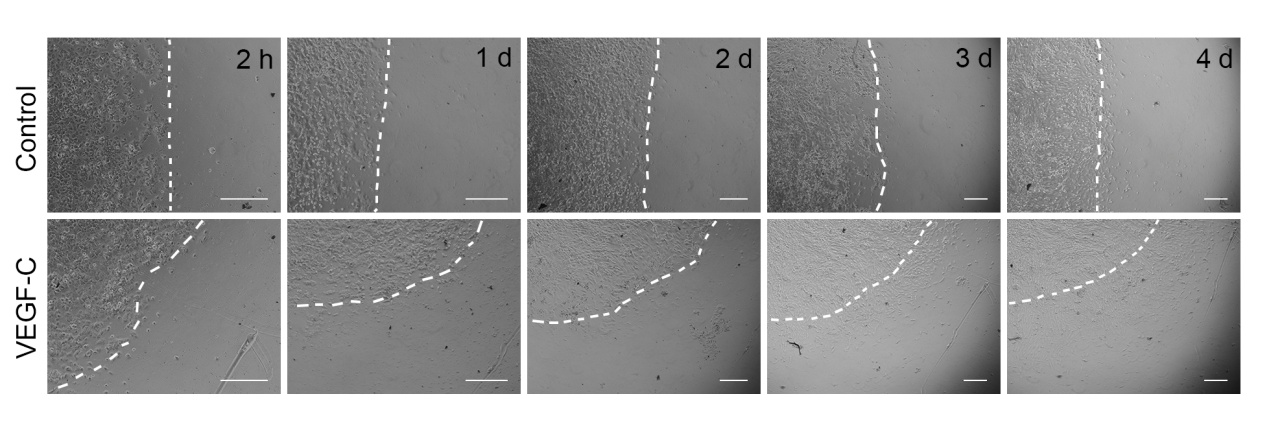
**

**Supplemental Fig. 1** The cells migrated from SAP hydrogel. Scale bar = 200 μm.

**Supplemental Fig. 2**

**
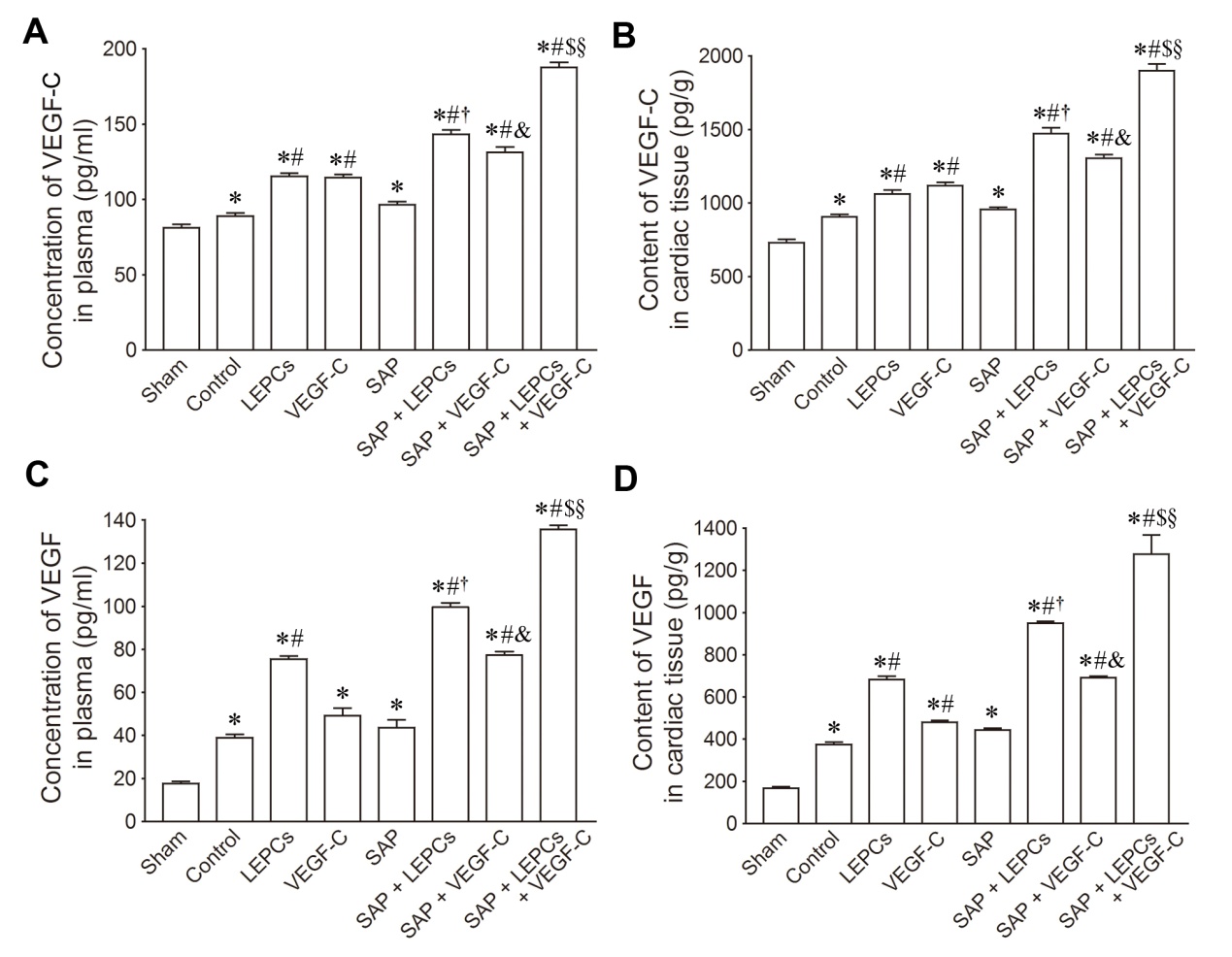
**

**Supplemental Fig. 2** The concentration of VEGF-C and VEGF in plasma and myocardium at 4 week after transplantation. **a, b** The concentration of VEGF-C. **c, d** The concentration of VEGF. **p* < 0.001 versus sham group, ^#^*p* < 0.01 versus control group, ^†^*p* < 0.001 versus LEPCs group, ^&^*p* < 0.001 versus VEGF-C group, ^$^*p* < 0.001 versus SAP + LEPCs group, ^§^*p* < 0.001 versus SAP + VEGF-C group.

**Supplemental Fig. 3**

**
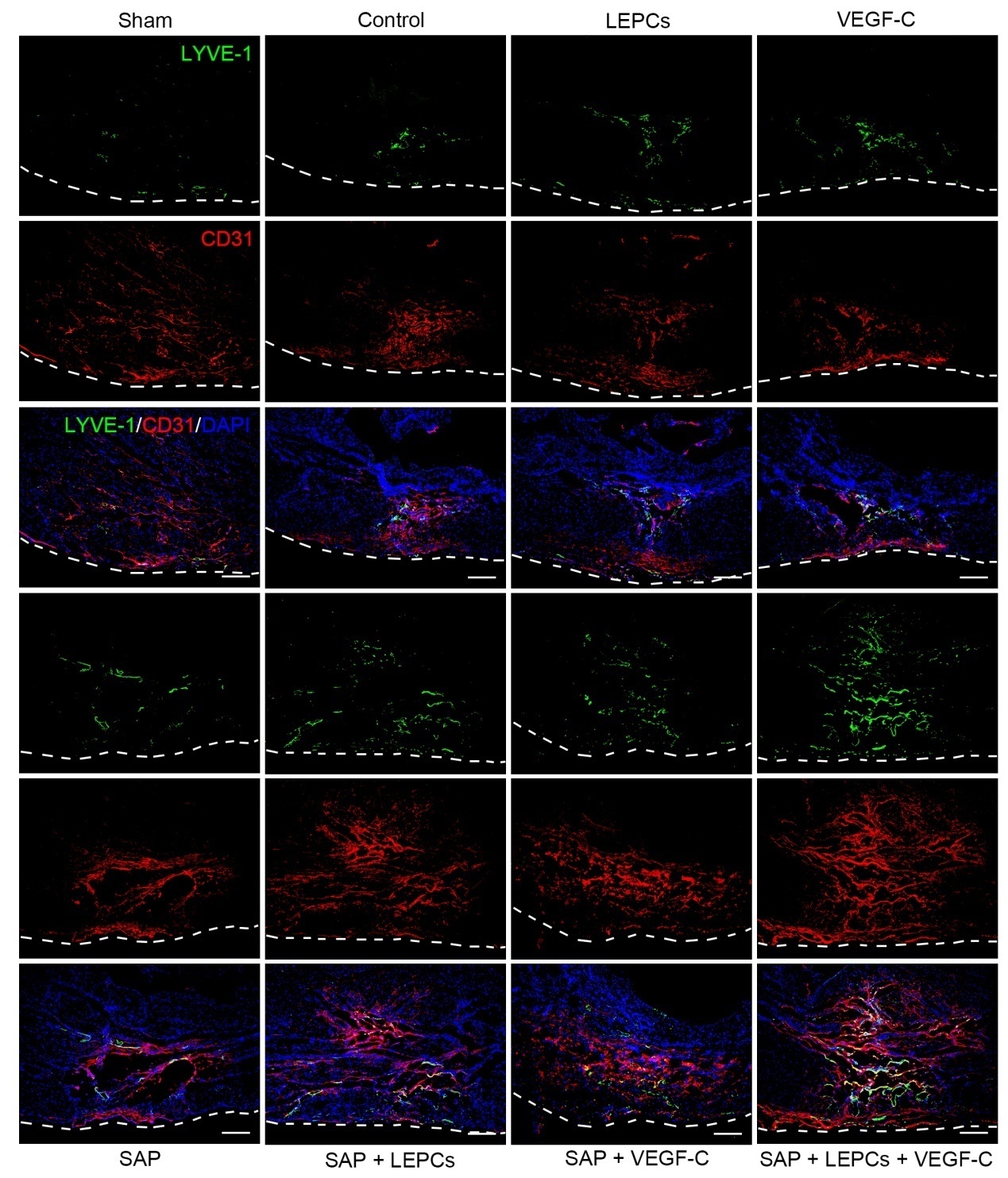
**

**Supplemental Fig. 3** The lymphatic vessels and microvessels at the peri-infarct region at 4 week after transplantation. The white dash line indicates the surface of the epicardium. LYVE-1 and CD31 immunostaining. Scale bar = 200 μm.

**Supplemental Fig. 4**


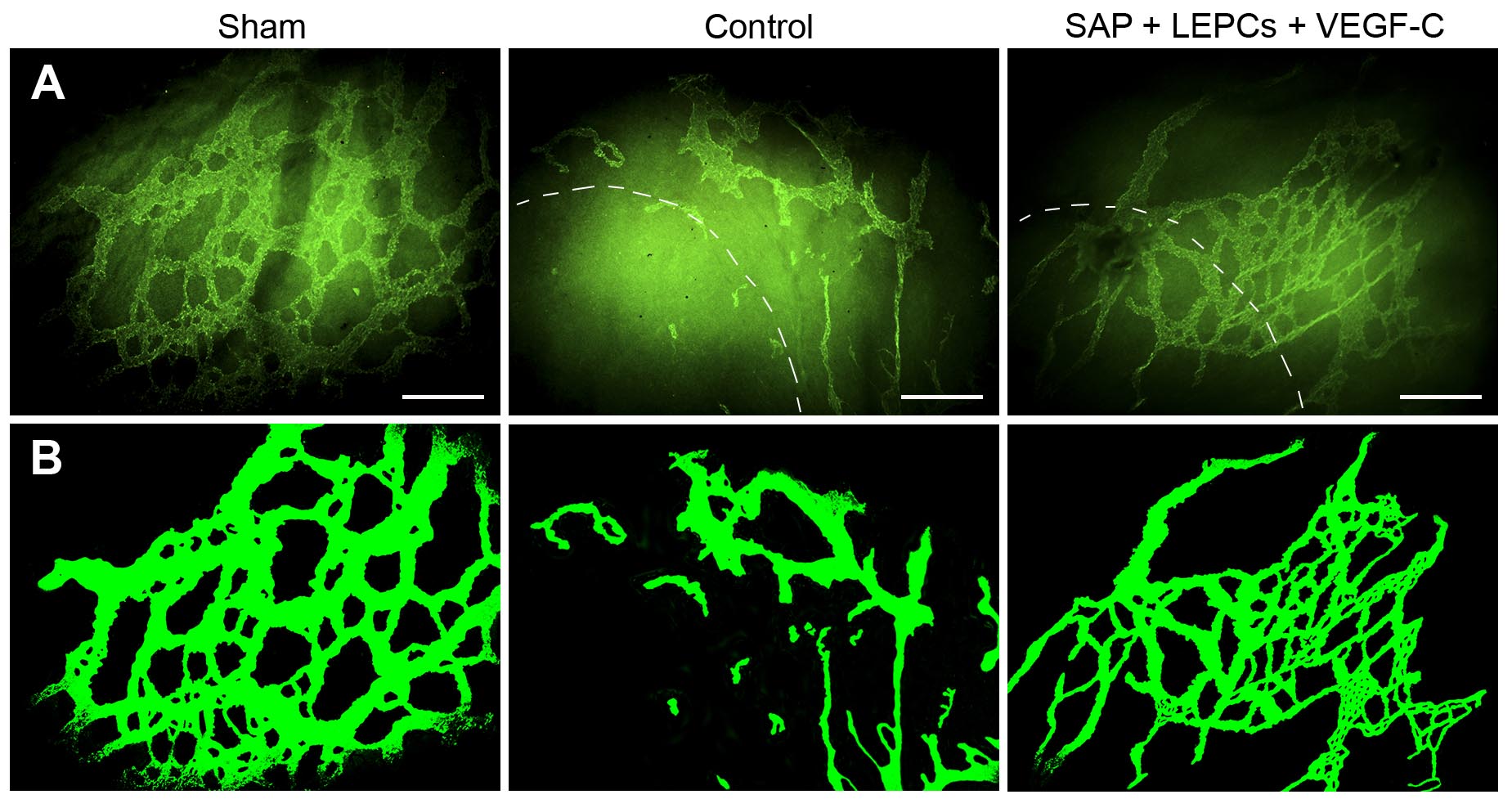


**Supplemental Fig. 4** The subepicardial lymphatic vessels at the peri-infarct and infarct regions at 4 week after transplantation. **a** The fluorescence microscopic images. LYVE-1 immunostaining. The white dash line indicates the border of the infarct region. Scale bar = 100 μm. **b** The reconstructed images from A with ImageJ software.
